# Auditory steady-state responses in school-aged children: a pilot study

**DOI:** 10.1186/s12984-015-0003-y

**Published:** 2015-02-10

**Authors:** Luciana Macedo de Resende, Sirley Alves da Silva Carvalho, Thamara Suzi dos Santos, Filipe Ibraim Abdo, Matheus Romão, Marcela Cristina Ferreira, Carlos Julio Tierra-Criollo

**Affiliations:** Speech Therapy and Audiology Department, Federal University of Minas Gerais, Belo Horizonte, Brazil; Institute of Biological Sciences, Federal University of Minas Gerais, Belo Horizonte, Brazil; Electrical Engineering Post Graduation Program, Federal University of Minas Gerais, Belo Horizonte, Brazil; Federal University of Ouro Preto, Ouro Preto, Brazil; Biomedical Engineering Program, Alberto Luiz Coimbra Institute of Graduate Education and Research in Engineering, Federal University of Rio de Janeiro, Rio de Janeiro, Brazil

**Keywords:** Auditory evoked potentials, Hearing, Electrophysiology, Hearing tests

## Abstract

**Background:**

The use of Auditory Steady-State Responses (ASSRs) for auditory screening in school-aged children, particularly in children who are difficult to test and children with disabilities, has not been explored yet. This pilot study investigated the use of ASSR for auditory screening in school-aged children.

**Materials and methods:**

A cross-sectional pilot study of 23 children aged 9 to 11 with normal-hearing thresholds and seven age-matched children with permanent moderate-to-profound bilateral hearing loss were examined. The tested carrier frequencies were 500, 1,000, 2,000, and 4,000 Hz, and the stimulus was modulated between 77 and 107 Hz. The ASSRs decreased according to the tested intensity levels of 50, 40, and 30 dB sound pressure level (SPL). Sensitivity and specificity were estimated from the responses of the children with normal hearing and those with hearing loss.

**Results:**

For the children with normal hearing, the 2,000-Hz frequency was detected more often in both ears and at all intensity levels compared to the other frequencies. The 500- and 2,000-Hz frequencies resulted in different response patterns in both ears. The time until response detection increased in parallel with amplitude reduction, as expected. The overall time required for the test was 15 minutes, including the time spent in volunteer preparation. The sensitivity was 97% for the three intensities, and the best specificity value was 100%, which was observed at 50 dB.

**Discussion:**

The response analysis indicated that a screening protocol for school-aged children could include 1,000, 2,000, and 4,000 Hz and that the recording of ASSRs was highly sensitive to internal and external factors. Fifty dB SPL should be considered a cut-off criterion for screening purposes because this was the intensity level with a sensitivity of 97% and a specificity of 100%.

**Conclusion:**

The use of ASSRs might be particularly useful in school-aged children who have difficulty performing subjective hearing tests. The sensitivity and specificity data suggested that the use of ASSRs was feasible as an auditory screening tool. In order to determine a protocol for screening, future studies should include a larger sample and children with mild hearing loss.

## Background

An evoked potential is defined as electric activity that is generated by the brain in response to exposure to a sensory stimulus [1,2]. Auditory evoked potentials are divided into transient and auditory steady-state responses (ASSRs) [1]. The high stimulus rate of auditory stimuli in ASSRs causes the response to a given stimulus to overlap with the response to a subsequent one [2]. Amplitude-modulated (AM) tones are widely used to record these responses [3,4].

The events in the cochlea following exposure to AM tones are the origin of steady-state responses. AM tones induce vibration in the portion of the basilar membrane that corresponds to the carrier frequency, which excites the local inner hair cells (IHCs) [3]. The IHCs depolarize when stereocilia deflect in one direction, which rectifies the AM tone in the cochlea, and which is followed by low-pass filtering in the afferent auditory pathways to complete the process of demodulation, thus resulting in a spectral component with a value corresponding to the modulation frequency [3]. This mechanism allows the maximum firing of the auditory pathway fibers to synchronize with a modulating wave phase [1]. Electroencephalography (EEG) detects the rectified signal, which is used as a reference in the investigation of an individual’s hearing threshold.

Afferent auditory pathways exhibit tonotopic organization. Therefore, AM tones trigger responses in specific regions along the auditory pathway depending on the stimulus carrier frequency. Several studies [1,4-6] have investigated the sources that generate ASSRs. The responses to tones with low modulation frequencies (i.e., below 70 Hz) are mainly generated in the auditory cortex contralateral to the stimulated ear [4], but responses to higher modulation frequencies (i.e., frequencies above 70 Hz) have a subcortical origin [1,5,6]. Therefore, ASSR recordings assess the status of the auditory pathway up to the response-generating site [6-9].

Stimulus properties influence the response. An increase in tone intensity increases the response amplitude. A reduction in tone intensity reduces the response amplitude and increases response latency [10]. The stimulus carrier frequency also influences ASSR recordings because the auditory pathway that is specific to each carrier frequency responds in a particular manner. A study that applied high-intensity tones (85 dB Hearing Level) observed responses with greater amplitude to low frequency tones [10]. This phenomenon occurs because high-intensity auditory stimuli do not saturate the amplitude of the brainstem-generated responses when the carrier frequency is low, which is the opposite of the effects of high frequencies [10]. Areas anterior to the ones stimulated at low frequencies may become excited with stimuli with larger amplitudes [11]. Another difference in the carrier pathways is the increase in phase delay that parallels the intensity reduction, which is greater at lower frequencies [10]. The area of peak wave excitation approaches the apex as the frequency decreases, which increases the time needed to reach the maximum response.

A review [12] examined associations of the rate/velocity of nerve fiber responses with fiber synchrony, threshold amplitude, response saturation, and neural adaptation, and the results were consistent with the correlation of neural responses to auditory stimuli with neurophysiological notions. The review results indicated that thresholds are lower, less synchronized, and more influenced by adaptation when nerve fiber responses are faster compared to fibers with medium or slow response speeds.

Some studies have identified clinical applications for ASSR. ASSR can determine the function of the tonotopic characteristics of the auditory pathway because the stimulation and response recordings may be performed dichotically and multiple frequencies may be tested simultaneously [13]. ASSR is a tool that is relevant for diagnostic testing, and it is particularly useful in the early diagnosis of hearing loss in infants and newborns. Early diagnosis in this population is highly significant because normal hearing sensitivity is directly related to the adequate development of speech and language [14,15]. According to some studies, ASSR is able to identify residual hearing that cannot be detected with other electrophysiological tests in audiological examinations [12,16]. Another advantage of ASSR is that the detected thresholds are frequency specific, which provides information for hearing aid selection and fitting, especially in infants and small children. Although frequency-specific responses can be obtained with auditory brainstem responses as well as cortical responses, ASSRs are faster than these techniques because of the simultaneous stimulation and recording.

However, the use of ASSR for auditory screening in school-aged children, particularly those children that are difficult to test and children with disabilities (e.g., autism and developmental global delay), has not been explored. Some recommendations advise the use of audiometric measures in school-aged children and suggest the advantage of objective measures for screening purposes, especially impedance audiometry [17-19]. Objectivity in the execution and interpretation of ASSR is a defining feature. The reduced need for patient participation and examiner interpretation increases the reliability of the results. Therefore, the present study investigated and described ASSRs in order to develop a protocol for its use in the auditory screening of school-aged children, with the aim of having a screening tool that is feasible for all children.

## Methods

The present study was a pilot study that was conducted with a convenience sample and a cross-sectional descriptive design. The Federal University of Minas Gerais ethics committee approved this study under ruling no. 0369.0.203.000-10. In addition, the school where the data were collected authorized the research.

The sample included 26 children with ages from nine to 11 years who attended a private school, exhibited normal hearing according to pure tone and impedance audiometry, and had no hearing or balance complaints. Seven children with ages between 7 to 14 years with moderate to profound bilateral sensorineural hearing loss were included in the study. All of the participants signed an informed consent form according to the institutional ethics committee recommendations. Children with disorders of the external and/or middle ear were excluded from the study.

Clinical questionnaires collected information on the children’s prenatal, perinatal, and postnatal history and identified indicators of auditory risk and behaviors denoting the current state of auditory health and balance. The children’s parents completed the questionnaires and returned them to the investigators.

The following procedures were performed: anamnesis, external ear canal inspection, pure tone and impedance audiometry, and ASSR assessment. Visual inspection of the external ear canal established whether the external ear conditions allowed for proper test performance (i.e., excluded the presence of an obstruction or other conditions). Pure tone audiometry was used to establish the children’s auditory acuity in a soundproofed room with a clinical audiometer (Eymasa, Barcelona, Spain) with ANSI S3.6/ISO 389 calibration standards. Frequencies between 250 and 8,000 Hz were tested, and the results were interpreted with Bureau International d’Audio Phonologie (BIAP) criteria. Impedance audiometry assessed middle ear conditions with an impedance audiometer (model At235h, Interacoustics a/s, Assens, Denmark) with ANSI S3.6/ISO 389 calibration standards. A 226-Hz probe was introduced into the participants’ ears to capture tympanic membrane movement in response to pressure variations in the external ear. The contralateral and ipsilateral stapedius reflex was assessed at 500 to 4,000 Hz. The tympanograms were classified according to that described by Jerger [20], and the stapedius reflex was interpreted according to that described by Jerger and Jerger [21].

The ASSRs were recorded with an AudioStim system (NEPEB/UFMG, Belo Horizonte, Brazil). Before the ASSR recordings were made, the equipment was calibrated in a specialized laboratory according to existing norms. To check the AudioStim stimulus levels, an artificial ear was used (4152 model, Brüel & Kjær Sound & Vibration Measurement A/S, Nærum, Denmark) that was coupled to a sound level meter (2260 model, Brüel & Kjær Sound & Vibration Measurement A/S) [22]. Tests were performed in a sound-proofed room, and background noise was monitored with a sound pressure level (SPL) meter according to the earphone manufacturer’s recommendations. Responses were captured with silver chloride electrodes that were placed on the participants’ scalps in the following positions: Fpz (grounding electrode), Cz (active electrode), and at the nape of the neck just below the hairline (reference electrode). The children were instructed to remain quiet and still with their eyes closed during the test. Most of the tests were performed while the children slept naturally.

Dichotic stimulation and multiple frequencies were used. The following carrier frequencies were used: 500 Hz, 1,000 Hz, 2,000 Hz, and 4,000 Hz. The respective modulation frequencies included 77.15 Hz, 86.91 Hz, 98.63 Hz, and 104.49 Hz in the right ear and 81.05 Hz, 94.73 Hz, 100.59 Hz, and 106.45 Hz in the left ear. The stimuli were applied through inserted earphones (model 5A, Aearo Technologies, Indianapolis, IN, USA).

The ASSRs followed a decreasing order whereby the intensity levels of 50, 40, and 30 dB SPL were tested without interruption. When a lower intensity induced a response but a higher intensity did not, the higher intensity was retested after completion of the protocol. If no response was detected at any frequency with 50 dB SPL, a 60-dB SPL intensity was also tested.

The stimulus levels were chosen to determine a pass-fail criterion for the development of the screening protocol. Children with normal hearing and those with hearing impairment were evaluated in order to validate the protocol.

The responses were detected with magnitude-squared coherence (MSC) at a 5% significance level [23]. Each sweep lasted 1,024 s, and the maximum average was 480 sweeps. The artifact rejection amplitude was 15 μV. The individual EEG signal gain was 50,000, and filtering was set at 30 Hz and 300 Hz. After preprocessing, an algorithm for the detection and removal of artifacts analyzed the collected registers before the objective response detection (ORD) technique was applied.

Artifact removal consisted of segmentation of the EEG signals into 1,024-s sweeps that were individually analyzed and dismissed when amplitudes above 15 μV were found in at least 1% of each sample. Occasional removal of one or more sweeps did not compromise the records’ stationarity once the modulation frequencies were chosen in a way that a whole number of oscillations existed every 1,024 s.

The ORD technique used the MSC at a 5% significance level [23]. In the absence of a stimulus, the MSC values tended to approach zero. However, when an equal response occurred in all of the sweeps, the MSC values tended to one [23]. In order to consider the data a response, the MSC values had to be superior to the critical value for five consecutive sweeps.

Comparisons of the frequencies and intensity levels were performed with the Friedman test at a 5% significance level, and posthoc analyses were performed with the Tukey-Kramer multiple-comparison test. Sensitivity and specificity were estimated with a pass-fail criterion that was similar to the recommended school screening protocol in tonal audiometry of response presence in reaction to 1,000 Hz, 2,000 Hz, and 4,000 Hz [17].

## Results

A total of 26 hearing children were assessed, but three children were excluded due to methodological problems during data collection, including electrode displacement. Therefore, the data of 23 participants (11 males and 12 females) were analyzed. Both ears were assessed in all of the participants. The 60-dB SPL intensity was assessed in seven volunteers, 50 dB SPL was reassessed in nine, and 40 dB SPL was reassessed in eight. Seven hearing-impaired children were also assessed. All of them had bilateral permanent sensorineural hearing loss with varying degrees from moderate to profound hearing loss.

**Figure 1:**
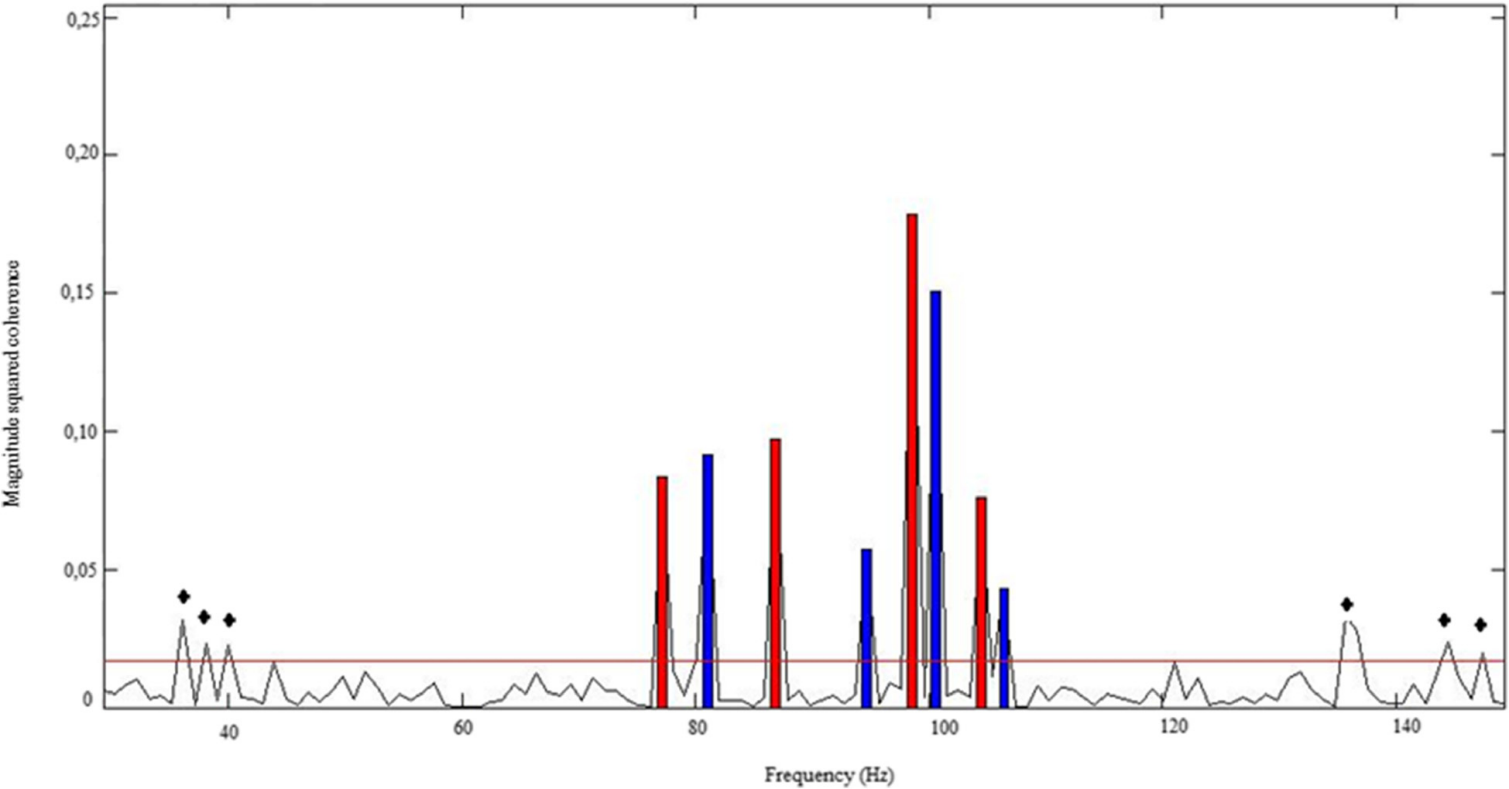
**Fifty-dB sound pressure level (SPL) auditory steady-state response (ASSR) detection with magnitude-squared coherence (MSC).** Individual #3 window detection: 50-dB SPL record. The MSC values after 150 sweeps are shown vertically. Horizontal Line: critical MSC (critical value to consider frequency presence). Bars: left ear (LE) and right ear (RE) modulations. The black star points indicate the frequencies with superior critical values after 150 sweeps of false positive detections.

A grand average of 50 dB SPL records is shown in Figure 1 from individual number 3 after 150 accepted sweeps. Please note that the detection of false-positive numbers (* indicated) was kept to 5%.

Correlations of the tested intensity levels and the number of detected frequencies showed parallel reductions. The 2,000-Hz frequency was detected more often in both ears and at all intensity levels compared to the other frequencies.

**Figure 2:**
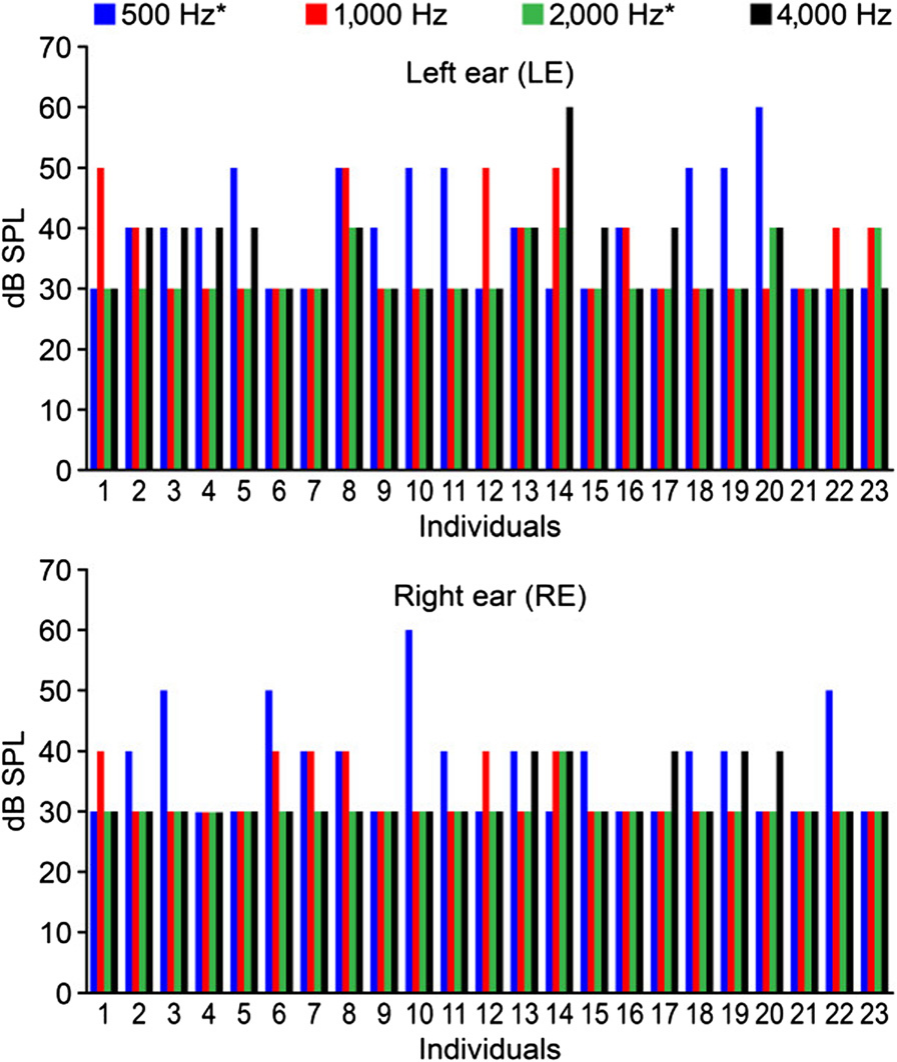
**The thresholds for each frequency in the individuals with normal hearing thresholds for the right and left ears.** The frequencies indicated with an asterisk (*) differ in the posthoc test with the Tukey-Kramer multiple comparison correction (p < 0.05).

The response detection time tended to increase in parallel with the intensity decrease (Table 1). At 30 dB SPL, all of the records were interrupted after only 480 scans (maximum stablished). For the individuals with hearing loss, all of the records were interrupted after only 480 sweeps (maximum stablished). Figure 2 depicts the lowest intensity in which 500 Hz, 1,000 Hz, 2,000 Hz, and 4,000 Hz were detected in the right and left ears in each individual with normal hearing. The Friedman test revealed significant differences between the detected frequencies in the right (p = 0.003) and left (p = 0.019) ears. Posthoc tests of the frequencies in both ears that were performed with the Tukey-Kramer multiple-comparison test showed that the lowest intensity of detected responses differed between 500 Hz and 2,000 Hz in both ears (indicated with an asterisk in Figure 1), which indicated that a 2,000-Hz response was present at significantly lower intensities compared with a 500-Hz response.

**Table 1:** **Average detection time per intensity**

**Intensity**	**Mean**	**SD**
50 dB	4.41	2.76
40 dB	5.44	2.30
30 dB	8	-

Time is measured in minutes; SD = standard deviation.

Table 2 show the true-negative and true-positive results for the pass/fail criterion of the presence of responses in reaction to 1,000 Hz, 2,000 Hz, and 4,000 Hz in each stimulus level. The analysis considered 46 normal-hearing ears and 14 hearing-impaired ears. True-negatives were more frequent in protocol 1 (n = 45), but, in protocol 3, only 21 true negatives were observed. However, for the three proposed protocols, the true-positive numbers were 14. Therefore, the estimated specificities were 100% for all of the intensity protocols. The sensitivity was 97% for protocol 1 (50 dB), 83% for protocol 2 (40 dB), and 46% for protocol 3 (30 dB).

**Table 2:** **Description of the simulated protocols for the left ear**

**Protocols**	**Stimulus level**	**Pass/fail criteria**	**True-positives (TP)**	**True negatives (TN)**	**Specificity**	**Sensitivity**
Protocol 1	50 dB SPL	1,000 Hz, 2,000 Hz, 4,000 Hz present	45	14	100%	97%
Protocol 2	40 dB SPL	1,000 Hz, 2,000 Hz, 4,000 Hz present	38	14	100%	83%
Protocol 3	30 dB SPL	1,000 Hz, 2,000 Hz, 4,000 Hz present	21	14	100%	46%

(1 to 3: first column), stimulus level (second column), and pass-fail criteria (third column). The fourth column shows the calculated true negatives in the 46 ears with normal hearing thresholds. Column 5 shows the true-positive results in the 14 ears with moderate to profound hearing loss. Columns 6 and 7 show the sensitivity and specificity results. The Objective Response Detection (ORD) technique was MSC.

## Discussion

No consensus on the inclusion of ASSRs as a tool for audiological diagnoses is apparent in the literature. The usefulness of ASSRs is acknowledged, but the literature emphasizes the use of ASSR with other tests [24], primarily because of the difficulty in establishing correlations between the electrophysiological thresholds and pure tone audiometry, which is the gold standard for audiological diagnosis [25].

### Stimulus level

The protocol that was formulated for the present study included assessments of three intensity levels (50 dB SPL, 40 dB SPL, and 30 dB SPL). The 60-dB SPL frequency was only tested in cases who exhibited no response to 50 dB SPL, which occurred in seven participants. The 50-dB SPL and 40-dB SPL intensities were reassessed in 17 participants in whom a lower intensity level induced a response while the higher intensity did not.

Some studies [11,26] have shown that steady-state electrophysiological thresholds vary from 15 dB SPL to 50 dB SPL as a function of the carrier frequency, investigated population, and experimental protocol in normal-hearing individuals. No references in the literature account for the lack of response to some frequencies within this intensity interval. Therefore, future studies are needed to investigate these findings more thoroughly.

The descriptive analysis suggested two tendencies in response detection relative to the factor intensity. The number of responses detected decreased and the time to detection increased in parallel with stimulus intensity reductions, as expected (Table 1). Please note that, at 30 dB SPL, no standard deviation was observed because the entire record time was used. This behavior was expected because low-intensity sounds behave as modifying variables that reduce response amplitude and increase response latency [10].

The individual response thresholds at each frequency for the normal-hearing children (Figure 1) varied from 40 to 30 dB SPL. This finding partially agreed with the literature. One study [27] found a 90% response in all frequencies from 50 to 40 dB SPL. The definition of a cut-off point for a stimulus intensity is relevant because the aim of the present study was to gain information to develop a screening protocol. The literature suggests the poor reliability of ASSR responses in individuals with mild hearing loss [28]. Therefore, we suggest the use of 50 dB SPL as the cut-off point for screening with the Audiostim system protocol in order to avoid the occurrence of false-negative results.

### Carrier frequency

Multiple comparisons were performed relative to each ear. A difference was found between 500 Hz and 2,000 Hz in both ears, and the highest thresholds corresponded to 500 Hz (Figure 1). These findings further suggested the influence of environmental conditions on the reliability of the results. Previous studies have found 500 Hz to be the worst frequency [27,29-32]. Low frequencies are more affected by the increased response latency and phase delay that parallel the intensity reduction compared to high frequencies [8]. Therefore, 500 Hz was expected to exhibit the poorest result of the tested frequencies.

Our finding that 2000 Hz provided the lowest thresholds was supported by the findings of previous studies. [29,31]. However, our results disagreed with the previous results that 4,000 Hz exhibited the lowest thresholds [27]. However, higher frequencies seemed to show the lowest thresholds, despite the discrepancy. Fibers in the areas that correspond to the high frequencies are likely to be fast-response fibers that are associated with lower thresholds based on the rate/velocity of the various nerve fiber responses (spontaneous rate) [12]. The opposite characteristics of 500 Hz are probably associated with slow-response fibers.

### ASSRs in school-aged children: protocol screening

The aim of auditory screening in school-aged children is the identification of hearing losses that might affect learning and the ability to read and write and impair overall school performance in addition to the implementation of early intervention programs [33]. The adequate perception of speech sounds is essential for the development of language and satisfactory literacy skills [34]. ASSRs might be particularly useful in children with difficulty performing subjective hearing tests.

Appropriate speech perception requires positive responses to high frequencies (above 1,000 Hz) because these frequencies correspond to the sound of most consonants, which account for 60% of speech intelligibility [28]. Low-frequency sounds and environmental noise contribute to sound localization skills [35], which are relevant for the proper execution of daily living activities. The analysis of response detection per frequency found significant differences between the low and high frequencies that were investigated in the present study. The 500-Hz frequency was detected the least. Moreover, the gold-standard protocol for school screening consists of subjective audiometric testing of 1,000, 2,000, and 4,000 Hz [17,36]. In this pilot study, a similar protocol was adopted and tested with an objective assessment tool.

The time needed for response detection at a single intensity level was six min on average. The overall time that was required for the test was 15 min, including the time spent in volunteer preparation (e.g., electrode and earphone placement and instructions). This period of time is close to the time that is required for pure tone audiometry, which supports the feasibility of ASSR as a screening tool. In addition, the objectivity in the execution and interpretation of the ASSR, its ability to detect frequency-specific hearing thresholds, and the possibility to assess children of any age, including those who are difficult to test and children with special needs, should be emphasized.

It must be pointed out that the sensitivity and specificity that were obtained for protocol 1 (50 dB) were adequate according to the American Speech Language and Hearing Association guidelines for school screening [17]. Nonetheless, the inclusion of children with moderate to profound hearing losses may account for the 100% specificity. It is necessary to include individuals with mild hearing loss in a future study in order to establish the feasibility of this technique and protocol. It would also be advisable to collect information from test and retest, which was a limitation feature in this study.

## Conclusions

The results showed that 500 Hz and 2,000 Hz exhibited different response patterns in both ears of children with normal hearing. The time for response detection increased in parallel with amplitude reduction.

The responses analysis indicated that a criteria for a screening protocol of school-aged children could include 1,000 Hz, 2,000 Hz, and 4,000 Hz with a cut-off point of 50 dB SPL for the stimulus intensity. The total time required for the protocol was 15 min.

Future studies should be conducted of larger samples as well as children with mild hearing loss, so that the sensitivity and specificity analysis of the suggested protocol can be fully established.
